# Rosmarinic Acid and Related Dietary Supplements: Potential Applications in the Prevention and Treatment of Cancer

**DOI:** 10.3390/biom12101410

**Published:** 2022-10-02

**Authors:** Jiachao Zhao, Liwei Xu, Di Jin, Yu Xin, Lin Tian, Tan Wang, Daqing Zhao, Zeyu Wang, Jing Wang

**Affiliations:** 1 College of Integrated Traditional Chinese and Western Medicine, Changchun University of Chinese Medicine, Changchun 130117, China; 2 Department of Respirology, First Affiliated Hospital to Changchun University of Chinese Medicine, Changchun 130021, China; 3 College of Chinese Medicine, Changchun University of Chinese Medicine, Changchun 130117, China; 4 School of pharmaceutical sciences, Changchun University of Chinese Medicine, Changchun 130117, China; 5 Northeast Asia Research Institute of Traditional Chinese Medicine, Changchun University of Chinese Medicine, Changchun 130117, China

**Keywords:** rosmarinic acid, cancer, tumorigenesis, adjuvant therapy, molecular mechanism

## Abstract

Cancer constitutes a severe threat to human health and quality of life and is one of the most significant causes of morbidity and mortality worldwide. Natural dietary products have drawn substantial attention in cancer treatment and prevention due to their availability and absence of toxicity. Rosmarinic acid (RA) is known for its excellent antioxidant properties and is safe and effective in preventing and inhibiting tumors. This review summarizes recent publications on culture techniques, extraction processes, and anti-tumor applications of RA-enriched dietary supplements. We discuss techniques to improve RA bioavailability and provide a mechanistic discussion of RA regarding tumor prevention, treatment, and adjuvant therapy. RA exhibits anticancer activity by regulating oxidative stress, chronic inflammation, cell cycle, apoptosis, and metastasis. These data suggest that daily use of RA-enriched dietary supplements can contribute to tumor prevention and treatment. RA has the potential for application in anti-tumor drug development.

## 1. Introduction

Cancer is a significant public health problem worldwide. Diagnostic technologies and treatments, including surgery, targeted therapies, and immunotherapies, have made significant advancements in the past 30 years. The risk of cancer death has dropped by 32%; however, the progression of advanced tumors, post-treatment drug resistance, and recurrence remain the most critical aspects of clinical oncology [1]. Potential challenges, long-term treatment, and repeated hospitalizations severely impact the quality of life, substantial financial burden, and psychological stress [2]. Studies showed that some dietary supplements, ethnic herbs, and teas are used for tumor prevention and treatment [3,4,5]. Some natural products from diets or plants are potential anti-tumor drugs and chemotherapy sensitizers [6].

**Figure 1:**
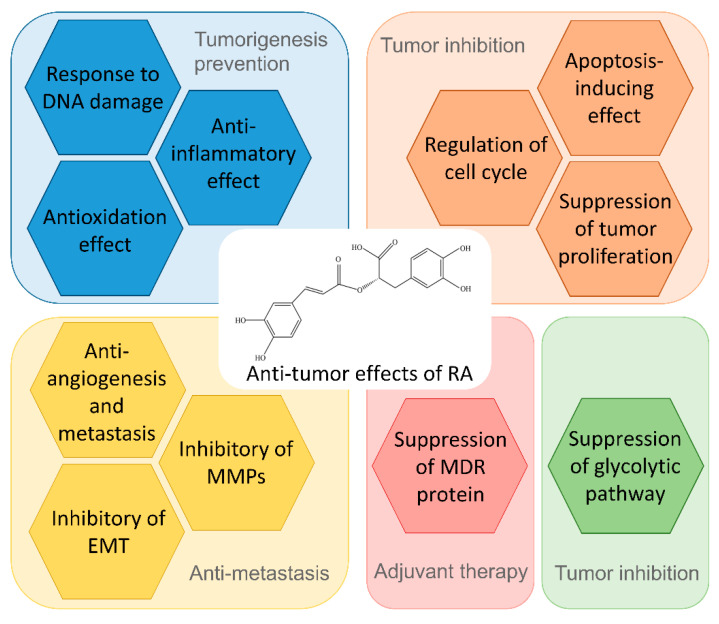
Summary of anti-tumor effects of RA.

Rosmarinic acid (RA) is a flavonoid commonly found in plants in the Lamiaceae family. RA-rich plants such as *Perilla frutescens* (L.) Britton, *Rosmarinus officinalis* L., and *Melissa officinalis* L. are popular worldwide and used in tea, herbs, cooking condiments, spices, and fruits. RA is used to improve health because of its nutritional properties and has been noted to have potent antioxidant activity [7,8]. In the past ten years, it has been noted that these plants might prevent and treat tumors. Isolation of the anti-tumor components of the plant revealed that the active components include polyphenols. Studies found that RA can prevent tumorigenesis, inhibit tumor growth, and sensitize chemo-radiotherapy agents as adjuvant therapy [9,10,11]. The preparation of RA depends on the purification after biosynthesis of plants, and the recent research proposes synthesizing RA in vitro by engineering bacteria [12]. The bioavailability of RA is low; therefore, the improvements of the dosage form and the development of chemical delivery systems are necessary for anti-tumor applications [13]. This review summarizes the anti-tumor applications, extraction processes of RA-rich plants, and anti-tumor mechanisms to provide in-depth mechanistic insights. This review aims to provide the latest evidence on the biological properties and anti-tumor applications of RA and RA-enriched plants (Figure 1).

## 2. Methodology

A literature search was performed in PubMed and Google scholar from January 1998 to May 2022, and the last search date was 30 April 2022. The search term was “rosmarinic acid”. A secondary search was conducted by screening the list of articles that met the inclusion criteria. The keywords were “cancer” OR “tumor” OR “carcinoma” OR “malignancy”. The obtained 306 articles were screened, 31 review articles and 3 articles not published in English were removed. A further 175 relevant studies were excluded by reading the abstract, an additional 21 records identified as eligible articles. In total, 118 articles were sorted and classified. Finally, we organized the tables, wrote the text, and made figures to summarize the application of RA anticancer effects according to the SANRA and previously literature review [14,15].

## 3. Culture Techniques, Extraction Processes, and Anti-Tumor Applications of RA-Rich Plants

RA-rich plant extracts are functional ingredients and supplements that have become popular products in the health industry. Studies on the extraction processes and anti-tumor applications of RA-rich plants are summarized in Table 1.

*Rosmarinus officinalis* L. (rosemary) is a popular culinary herb worldwide and in European folk medicine. Aqueous extract of leaves inhibited the proliferation of cervical cancer, breast cancer, and T-cell leukemia cells [16]. Ethyl acetate extracts were enriched in RA, and they displayed antioxidant activity and promoted the apoptosis of colorectal cancer (CRC) cells [17]. The ethanol extract of rosemary dried leaves promoted apoptosis to enhance sensitivity to cisplatin (DDP) in ovarian carcinoma cells [18].

*Perilla frutescens* (L.) Britton is used as a medicinal plant in China, Japan, and Thailand. The aqueous extract of *P. frutescens* leaves are rich in RA and promote hepatocellular carcinoma (HCC) apoptosis by regulating apoptosis-related genes detected by cDNA microarrays [19]. Osakabe et al. optimized the extraction process of RA from leaves of *P. frutescens* with a concentration of 68% *w/w*. The extractive fraction and RA reduced inflammation and oxidative stress and reduced tumor size in skin cancers induced with 7,12-dimethylbenz[a]anthracene (DMBA) and 12-tetradecanoylphorbol 13-acetate (TPA) [20]. The seeds of *P. frutescens* are rich in omega-3 fatty acids and RA. After extracting oil from the seeds, RA can be enriched by 70% ethanol and ethyl acetate extraction. The RA-enriched fraction reduced reactive oxygen species (ROS) and inhibited invasion through the NF-κB pathway in A549 cells [21,22].

*Melissa officinalis* L. is a traditional herbal tea from the Mediterranean. Studies found that *M. officinalis* L. with ethanol extraction enriched RA [8,23]. These extracts had anti-tumor effects on HCT116 and H460 cells. The polyphenolic extract is a candidate for an antioxidant to protect human keratinocytes from UVB-induced skin damage [24]. Hydroxyphenylpyruvate reductase (*HPPR*) from *M. officinalis* L. was isolated and characterized as RA biosynthesis-related gene. Several terpenoid synthesis genes were identified and classified in this study [25].

*Ocimum basilicum* L. (i.e., basil) is consumed as a seasoning worldwide. Hosam et al. compared six cultivars of basils and found that basil leaf extracted from methanol had anti-tumor effects and was rich in RA [26]. Ethanol extract from basil leaves prevented metastasis in head and neck squamous cell carcinoma (HNSCC) [27]. In addition to the leaves, the callus of basil has high RA content. Saher et al. improved tissue culture technologies to increase RA production. They explored several plant growth regulators and found that 5 mg/L 6-benzylaminopurine (BAP) combined with 1 mg/L naphthalene acetic acid (NAA) yielded the best phenolic yield (346.08 mg/L), including 7.4 mg/g RA [28]. Subsequently, callus of basil grown on a medium supplemented with 10 mg/L CuO-NPs yielded the highest RA accumulation (11.4 mg/g) [29]. Light-emitting diode irradiation increased the RA content of callus 96.0 mg/g, 2.46-fold higher than control [30].

Extracts and processed products from *Origanum vulgare* L. are condiments in cooking, essential oils, and wine. It is a medicinal plant used to treat asthma, indigestion, headaches, and rheumatism in Turkey. Water-soluble ethyl acetate extract had antioxidant and anti-proliferative activities against C6 (rat glioma), and HeLa cells; RA, hesperetin, and hydroquinone were the active ingredients [31]. Juste et al. evaluated antioxidant and anticancer activities in various strains of *O. vulgare* and found that RA content was positively correlated with antioxidant activity [32].

Thyme is a perennial Lamiaceae herb native to temperate regions of Europe, North Africa, and Asia. It is used as a culinary seasoning and a medicinal plant in ethnomedicine. *Thymus vulgaris* L. callus crude extract (RA content 5.67 mg/g) inhibited human breast cancer cells [33]. Antioxidant and cytotoxic properties of *Thymus longicaulis* C. Presl were analyzed during various life cycle phases. Oct12 extract was rich in RA and showed a marked biological activity and cytotoxicity against several tumor cells [34].

The genus *Salvia*, also belonging to the Lamiaceae family, possesses anticancer medicinal properties. *Salvia officinalis* L. and *Salvia fruticosa* Mill. (Mediterranean medicinal plants) contain RA in aqueous extracts from 50 to 70 μg/mL. These extracts inhibited proliferation in breast cancer and colon cancer cell lines via the mitogen-activated protein kinase (MAPK)/ extracellular signal-regulated kinases 1 and 2 (ERK1/2) and the phosphatidylinositide-3-kinase (PI3K)/AKT pathways [35,36]. Research has shown that foliar spraying with NO and Si and under Cu stress in *S. officinalis* elevated total RA content by 2-fold above control leaves. The seedlings were irrigated with sodium silicate (1 mM Si), sodium nitroprusside (200 μM as a NO donor), and 200 μM CuSO_4_ [37]. *Salvia miltiorrhiza* Bunge is a popular Chinese medicinal herb. Methyl jasmonate (MeJA) enhanced the synthesis of RA in *S. miltiorrhiza* through regulation of the transcription factor gene *SmMYB2* and secondary metabolism-related genes [tyrosine aminotransferase (*TAT*) and *HPPR*] [38,39]. Equal amounts of cellulase A and protamex mixture exhibited maximum effectiveness in extracting RA at 28.23 mg/g [40]. The callus culture of stem and leaf explants of *S. miltiorrhiza* promoted RA biosynthesis. RA and salvianolic acid B were cytotoxic primary phenolic compounds for acute lymphoblastic leukemia (ALL) cells [35]. Sage tea made from *Salvia* helps prevent colon cancer by inhibiting oxidation and DNA damage [41].

*Prunella laciniata* (L.) L. is a plant of the Labiaceae family that has been used as food and medicine in China for thousands of years. A tyrosine aminotransferase of *Prunella vulgaris* (*PvTAT*) is an RA biosynthesis enzyme applicable to engineering natural products [42]. Studies showed that 60% ethanol extract of *P. laciniata* showed high antioxidant activity in vitro and in vivo and inhibited tumor load in tumor-bearing C57BL/6 mice [43,44].

In addition, RA is the primary active ingredient in several plants. In *Gastrocotyle hispida* (Forssk.) Bunge, grown in Saudi Arabia, RA was a potent anti-breast cancer and anti-HCC active component [45]. The ethyl acetate fraction extract of *Glechoma hederacea* L. promoted mitochondrial membrane potential destruction and apoptosis in HCC cells. Substant polyphenols, including RA, caffeic acid, and ferulic acid, were separated using high-performance liquid chromatography [46]. RA is the principal polyphenol in *Ehretia tinifolia* L. and showed cytotoxicity and potent antioxidant activity against several cancer cell lines [47].

Plant culture techniques include the addition of nanoparticles, and the co-culture of plants and bacteria were used to increase RA yield. Young seedlings of *Leonotis nepetifolia* (L.) R.Br. were infected with *Rhizobium rhizogenes* strain A4. The dominant compounds in the extracts contained 2643 µg/g RA, which was 43% higher than in untransformed roots. The transformed roots extract showed better cytotoxic effects against breast cancer [HCC1937 cells the half-maximal inhibitory concentration (IC_50_) = 750 µg/mL] and leukemia (NALM-6 cells IC_50_ = 550 µg/mL), meanwhile, HUVEC normal cells had no change in cell viability at the same concentration [12].

Transformed roots of *Dracocephalum kotschyi* Boiss. were treated with 50 mg/L titanium dioxide nanoparticles (TiO_2_ NPs) for 24 h; this treatment raised RA levels to 530.5 μg/g by increasing the expression of *PAL* and *RAS* genes [48]. *D. kotschyi* was co-cultivated with *Agrobacterium rhizogenes* to mediate hairy root growth. Hairy roots were exposed to 75 mg/L Fe NP for 24 h, yielding RA content of 1194 μg/g [49]. 

The endangered plant species *Satureja khuzistanica* Jamzad (from Iran) yielded RA in methanol extracts ranging from 0.59% to 1.81%. Abbas et al. developed cell suspension cultures of *S. khuzistanica* supplemented with 100 μM MeJA as an elicitor to improve RA production to 3.9 g/L [50]. Subsequently, the authors found that suspension cultures treated with pre-optimized coronatine (1 µM) obtained 2.67 g/L RA production, and the crude extract induced apoptosis of MCF-7 cells [51]. These findings demonstrate the considerable potential of in vitro cell culture of plants to induce the biosynthesis of compounds for RA production.

Based on co-culture fermentation technology, RA is synthesized by fermenting plant pericarp and bacteria. Fresh grape skins were vacuum-cooled, powdered, and fermented by *Lactobacillus plantarum* KFY02 for 96 h. The fermentation broth was rich in RA, rutin, and resveratrol which have antioxidant and liver cancer inhibitory activities [52].

Synthetic biology has made rapid progress and shown broad application prospects in various fields. Engineering bacteria introduce genes into plants or other animals and uses known biochemical reactions in nature to produce small molecular compounds, primarily natural products. Several studies reported *de novo* synthesis of RA by engineered bacteria. Enzymes including rosmarinic acid synthase (RAS), 4-hydroxyphenlacetate 3-hydroxylase, D-lactate dehydrogenase, TAT, and tyrosine ammonia lyase catalyzed reactions for RA biosynthesis [53,54,55,56,57]. Yan et al. achieved 320.04 mg /L*h RA productivity from caffeic acid and 3,4-dihydroxyphenyllactic acid using an ATP and CoA cycle regeneration system [58]. Li et al. developed a three-strain co-culture synthetic route to produce RA from glucose; RA bioproduction reached 172 mg/L [59].

**Table 1:** The production process and anti-tumor effects of plants constitute a great source of RA.

Source	Biotechnological Application for Production and Extraction Process	RA Content	Anti-tumor Effect	Ref
*Rosmarinus officinalis* L.	Aqueous extract of leaves	45.64 mg/g	Cervical cancer Breast cancer T-cell leukemia	[16]
	Leaves removed the lipidic phase using hexane. Then, extracted in ethyl acetate	Approximately 50.11% *w/w* RA	Colorectal cancer	[17]
	Dried leaves of *Rosmarinus officinalis* L. were extracted with 70% (*v/v*) ethyl alcohol overnight at 22 °C on a shaker. The stock solutions were collected from the supernatant	-	Ovarian carcinoma	[18]
*Perilla frutescens* (L.) Britt.	Fresh Perilla leaves were extracted with 1% *w/v* citric acid at 90 °C for 30 min, then mixed with n-butanol, dried, and dissolved in water. Elution with 0.1% *w/v* TFA containing 80% *v/v* methanol on Diaion HP2MG column	68% *w/w* RA of freeze-dried powder	Skin carcinogenesis	[20]
	The dried leaves were chopped, boiled in 1 L of distilled water for 1 h, and filtered. The supernatant was lyophilized.	-	HCC	[19]
	The seed meal was extracted in 70% ethanol and dried, then dissolved in ethyl acetate	600.32–647.68 mg/g	Lung cancer	[21,22]
*Melissa officinalis* L.	50% ethanolic extracts of leaves	N.A.	Colorectal cancer	[23]
	Ethanolic extracts of dry leaves	184.4 ± 0.3 mg/g	Lung cancer	[8]
	Ethanolic extract	Approximately 18%	Photoaging and skin cancer	[24]
*Ocimum tenuiflorum* L.	Leaves were soaked in 95% ethanol for two weeks, then filtered and dried	Approximately 7.86 mg/g	HNSCC	[27]
*Ocimum basilicum* L.	99% methanol extracts of dry leaves contained RA 3.01 mg/g	3.01 mg/g	Cervical cancer Breast cancer T-cell leukemia	[26]
	Callus of basil supplemented with 5 mg/L BAP and 1 mg/L NAA and extracted using 100% ethanol	7.4 mg/g	-	[28]
	Callus of basil grown on medium supplemented with 10 mg/L CuO-NPs, then extracted using 99.9% methanol	11.4 mg/g	-	[29]
	Callus of basil grown on with LED irradiation (24 h, 660 nm), then extracted using methanol	96.0 mg/g	-	[30]
*Origanum vulgare* L.	The aqueous part of the plant was chromatographed on silica gel and eluted with hexane	0.15 mg/g RA/dry plant	Glioma Cervical cancer	[31]
	Herb was ground and sieved using a 125-μm sieve. The powder was extracted with hot reflux in 90% (*v/v*) ethanol at 95 °C for 4 h	Approximately 36 mg/g	Glioma Breast cancer	[32]
*Thymus vulgaris* L.	Dried callus was extracted by Soxhlet continuous extraction device	5.67 mg/g	Breast cancer	[33]
*Thymus longicaulis* C.Presl	The leaves were collected in October using 50% methanol for ultrasonic extraction	3.03 mg/mL	Leukemia Glioma Breast cancer Colorectal cancer	[34]
*Salvia officinalis* L. and *Salvia fruticosa* Mill.	Aqueous extracts	52.0 and 71.5 μg/mL RA of water extract	Colorectal cancer	[35]
*Salvia officinalis* L.	The seedlings were irrigated with 1 mM sodium silicate, 200 μM sodium nitroprusside, and 200 μM CuSO_4_	0.62 mg/g	-	[37]
*Salvia miltiorrhiza* Bunge	Ground powder was enzymatically incubated and extracted with Cellulase A, Protamex (1:1), and distilled water at 30 °C for 2 h with stirring.	28.23 mg/g	-	[40]
*Prunella laciniata* (L.) L.	60% ethanol extract of leaves	2.31 mg/g	Lung cancer	[43,44]
*Gastrocotyle hispida* (Forssk.) Bunge	80% methanol extracts from leaves	-	HCC Breast cancer	[45]
*Glechoma hederacea* L.	The whole plants were extracted in distilled water (3 hr at 100 °C) at a dilution of 1:50 (*w/v*), then extracted with ethyl acetate	174.10 ± 5.80 mg/g	HCC	[46]
*Ehretia tinifolia* L.	The juice in the fruit was applied onto an Amberlite XAD-7 column and eluted with methanol	-	Cervical cancer Breast cancer Colorectal cancer	[47]
*Dracocephalum kotschyi* Boiss.	Transformed roots were influenced by 50 mg/L tTiO2 NPs for 24 h exposure time and incubated for one week. The transformed roots were harvested and extracted under 80% methanol ultrasound	530.5 μg/g	-	[48]
	*In vitro* grown leaves were co-cultivated with *Agrobacterium rhizogenes* strain to mediate hairy root. Hairy roots were exposed to 75 mg/L Fe NP for 24h, then harvested and extracted under 80% methanol ultrasound	1194 μg/g	-	[49]
*Leonotis nepetifolia* (L.) R.Br.	Young seedlings were infected with *Rhizobium rhizogenes* strain A4, then harvested and extracted under 80% methanol ultrasound	2643 µg/g	Lung cancer Breast cancer T-cell leukemia	[12]
*Satureja khuzistanica* Jamzad	Cell suspension cultures of plants supplemented with 100 μM MeJA for 21 days Methanol extraction	3.9 g/L RA in cell suspension cultures	-	[50]
	Cell suspension cultures of plants elicited with 1 µM coronatine	2.67 g/L RA in cell suspension cultures	Breast cancer	[51]
*Lactobacillus plantarum*	Fresh grape skins were vacuum-cooled and powdered, fermented by *Lactobacillus plantarum* KFY02 for 96 h	-	HCC	[52]

## 4. Improvement of Bioaccessibility and Bioavailability—Novel Technologies

The pharmacokinetic profile of RA was summarized by Nunes et al.; the benefits of RA as a supplement are limited due to formulation challenges, bioaccessibility, and bioavailability [7]. Therefore, it is essential to improve the bioavailability of RA, including the improvement of pharmaceutical technology and developments of drug delivery systems. For toxicology, a dose of 169.6 ± 32.4 mg/kg in Kunming mice (6 weeks old) was shown to be lethal, indicating that RA was slightly toxic [60]. Meanwhile, clinical studies should be considered for further investigation. There are several clinical studies using RA-enriched dietary supplements. Among them, there were no reports of adverse reactions [61,62]; however, these cannot explain the anti-tumor effects and potential toxicity of RA for humans.

A study evaluating the bioavailability and nutrient kinetics of *Rosmarinus officinalis* L. phenolic compounds in healthy humans found that phase II derivatives of RA were RA-glucuronide, methyl-RA-glucuronide, dimethyl-RA-glucuronide, and dimethyl-RA, suggesting absorption in the small intestine [13]. The absolute oral bioavailability of RA butyl ester was 10.52%, compared to only 1.57% in its original form [63]. The absolute bioavailability of RA was improved to 89.63 % after pulmonary administration [64].

Veras et al. tested excipients for RA. Microcrystalline cellulose and polyvinylpyrrolidone have compatibility against physical interactions, chemical incompatibilities, high temperatures, and water [65]. Encapsulation techniques involved oligosaccharides (e.g., cyclodextrins), increasing solubility in aqueous environments. Complexation of RA with cyclodextrin improved antioxidant activity [65]. Several delivery systems of nanoparticles, solid lipid nanoparticles (SLN), and phospholipid complexes have been applied to improve the bioavailability and absorption of RA in the gastrointestinal environment. RA-loaded silk fibroin nanoparticles had better bioavailability and induced apoptosis of breast and cervical cancer cells in vitro [66]. A study reported RA’s dose safety and toxicity loaded into SLN composed of Witepsol and Carnauba waxes [67]. RA is encapsulated in a hydrophobic bilayer that enhances bioavailability when exposed to the gastrointestinal tract. Xue et al. developed iron-crosslinked RA–lipid conjugates with high contents of RA and doxorubicin (DOX), which had better stability, bioavailability, and synergistic anti-breast cancer efficacy [68]. RA–phospholipid complexes increased oral bioavailability through enhanced intestinal permeability; an in vitro assessment determined that it had better permeation and antioxidant activity [69].

## 5. Biological Processes and Mechanism of Action of RA in Tumor Prevention and Treatment

### 5.1. Antioxidation and Anti-Inflammatory Effect

Oxidative stress is caused by the excessive accumulation of free radicals and involves the development of aging, cancer, heart failure, brain damage, and immune disorders. Therefore, the daily consumption of vitamin-rich foods as non-enzymatic antioxidant supplements, or superoxide dismutase (SOD), catalase (CAT), and other health products as the supplements of antioxidant enzymes can effectively remove free radicals. Studies have shown that phenolic antioxidant RA had the function of scavenging free radicals, including ROS and H_2_O_2_, and enhanced antioxidant enzymes and non-enzymic antioxidants [70,71]. The antioxidant effect of RA is mainly related to preventing tumorigenesis and chemosensitization.

Long-term exposure to ionizing radiation and chemical carcinogens induces tumorigenesis. Ultraviolet (UV) exposure and administration of chemical carcinogens including DMBA, TPA, 1,2-dimethylhydrazine (DMH), and azoxymethane (AOM) were used as models of tumorigenesis. Increased metabolic activity in cancerous tissues generates high concentrations of ROS leading to pro-tumorigenic events [72]. RA exhibited a potent scavenging effect on ABTS and DPPH radicals and prevented skin and oral carcinogenesis [70,73]. RA enhanced SOD, CAT, and glutathione peroxidase (GPx) activities and reduced lipid peroxidation and cytochrome P450, significantly reducing DMH-induced intestinal polyps in vivo [74,75,76,77]. The accumulation of ROS is often accompanied by inflammation, and skin cancer and CRC are usually associated with long-term chronic inflammation and oxidative stress. RA enhanced nuclear factor erythroid 2-related factor 2 (Nrf2)/heme oxygenase-1 (HO-1) antioxidant system to downregulate NOD-like receptor family pyrin domain containing 3 (NLRP3) and interleukin-1β (IL-1β) in a skin carcinogenesis model caused by UVB radiation [78]. In AOM and dextran sulfate sodium-induced colorectal carcinogenesis animal models, interleukin-6 (IL-6) levels and progression of colitis-associated colon cancer were decreased by RA. The mechanism involved the downregulation of Toll-like receptor 4 (TLR4)/nuclear factor-κB (NF-κB) and signal transducer and activator of transcription 3 (STAT3) [9,79]. RA prevents colorectal carcinogenesis due to antioxidation and anti-inflammatory effects. RA is metabolized and absorbed by the intestinal epithelium, suggesting the utility of daily dietary RA supplementation [13].

The application of the antioxidant effect of RA in tumor therapy is adjuvant therapy and improving tumor side effects. The combination of blue light and RA for HNSCC decreased H_2_O_2_ production and inhibited epithelial growth factor receptor (EGFR) activation in vitro [80]. Free radical scavenging increased RA synergism with cytarabine (Ara-C) against leukemia cells [81]. In addition, the antioxidant activity of RA can improve the toxicity of anti-tumor therapy. The antioxidation properties of RA protected ovaries without attenuating the anti-tumor effect of cisplatin [71]. RA improved the hepatorenal toxicity induced by methotrexate and cardiotoxicity induced by DOX based on antioxidant activity [82,83,84].

The anti-inflammatory targets of RA in tumor therapy are cyclooxygenase-2 (COX-2) and NF-κB. RA inhibited COX-2 activity and downregulated ERK1/2 to exert anti-inflammatory effects in lung, breast, and liver cancer cells [85,86]. The molecular simulation predicted that Arg120 in COX-2 was the active site of RA [86]. RA induced apoptosis of acute leukemia, liver cancer, and breast cancer by inhibiting NF-κB-mediated inflammation [87,88,89]. Wu et al. found that RA targeted I-kappaB kinase-β (IKK-β) to inhibit the NF-κB signaling pathway using molecular docking [90]. Inflammatory factors, including tumor necrosis factor-α (TNF-α), IL-1β, IL-6, and transforming growth factor-β (TGF-β), were reduced after anti-tumor therapy with RA [86,88].

### 5.2. Response to DNA Damage

ROS accumulation and chronic inflammation lead to DNA damage and carcinogenesis [91]. RA inhibited DNA damage due to potent antioxidant capacity, which plays an essential role in preventing tumorigenesis. DMH-induced rat colorectal polyp model and UV-irradiated mouse skin model have verified that it attenuated DNA damage and inhibited tumorigenesis [24,92]. In the anti-tumor process, RA acts as a chemosensitizer in a ROS-independent manner to inhibit DNA damage repair, thereby negatively responding to DNA damage [90]. RA is used as adjunctive therapy to destroy DNA structure (with alkylating agents) and inhibit RNA and DNA synthesis (DOX drugs). RA was combined with alkylating agents in the treatment of CRC resulting in the suppression of DNA repair proteins [41]. The efficacy of DOX in HCC was amplified by combining with RA, which induced mitochondrial dysfunction and DNA damage [93]. Zhang et al. showed that LncRNA MALAT-1 was regulated by RA and promoted DNA damage in ovarian cancer cells; however, the mechanism remains unclear [94].

### 5.3. Regulation of Cell Cycle and Tumor Proliferation

Sustained unplanned proliferation is one of the hallmarks of cancer, characterized by the potentially infinite proliferation of cancer cells due to the uninterrupted cell cycle and cell division. Cell cycle-related inhibitors (cyclin-dependent kinases 4/6 inhibitors) arrest tumors in the G1 phase, thereby preventing proliferation; this mechanism has been applied in the treatment of several tumors [95]. RA induced cell cycle arrest in treating multiple tumor cells, mainly through upregulation of p53 and p21 and downregulation of cyclins D1, E, and B1 [96]. RA induced G0/G1-phase arrest in breast and pancreatic cancer [97,98]. G2/M arrest occurred in treating kidney cancer and oral cancer [99,100]. Cell cycle arrest represents an opportunity for cancer cells to enter apoptosis. RA increased the expression of apoptosis-related proteins, including BCL-2 associated X (BAX), caspase-3, and caspase-8, and attenuated the expression of anti-apoptotic proteins B cell lymphoma-2 (BCL-2) and poly (ADP-ribose) polymerase (PARP) [51,98]. The upstream mechanism of RA inducing cell cycle arrest included histone deacetylases 2 (HDAC2) and glioma-associated oncogene homolog 1 (Gli1). RA mitigated the restriction of HDAC2 on p53, thereby triggering cell cycle arrest [96]. RA enhanced proteasome-mediated degradation of Gli1 and inhibited the expression of downstream cyclin D1 and snail1 [98].

In addition to inducing cell cycle arrest to inhibit proliferation, RA can also directly regulate cell proliferation-related targets. The anti-proliferative ability of RA was improved at lower concentrations in combination therapy [11,101]. RA behaved the excellent anti-proliferative activity against HeLa, HT29, A549 and MCF6 cancer cell lines with the IC_50_ values of 249.80, 277.85, 241.47, and 220.25 µM [102]. EGFR is a primary target of the anti-proliferation effects of RA [80,101]. Virtual drug screening analysis revealed that RA selectively inhibited EGFR and spleen tyrosine kinase (SYK). Kai-Cheng et al. synthesized three RA derivatives against drug-resistant EGFR [103]. Microtubule affinity regulating kinase 4 (MARK4) controlled the early step of cell division. Mini-chromosome maintenance complex component 7 (MCM7) initiated eukaryotic DNA replication. RA bound to the active pockets of cell proliferation-related proteins MARK4 and MCM7 with better potency and inhibited protein functions in silico [104,105].

### 5.4. Apoptosis-Inducing Effect

RA increased the ratio of BAX/BCL-2, activated caspase family proteins, and inhibited PARP, leading to apoptosis in several tumor cell lines [51,96,106]. RA promoted caspase family proteins activity observed in different types of tumor cells in vitro, including CRC, lung cancer, oral cancer, glioma, osteosarcoma, and ALL [10,60,90,100,106,107,108]. PI3K/AKT is the primary pathway of RA-mediated apoptosis. In treating HCC and glioma, RA acted as a Fyn inhibitor, promoting the expression of apoptosis-related proteins through the PI3K/AKT and NF-κB pathways [10,109]. RA downregulated the PI3K/AKT/the mechanistic target of rapamycin (mTOR) signaling pathway to induce apoptosis and inhibited epithelial-mesenchymal transition (EMT) and tumor growth in HCC and osteosarcoma [108,110]. A study applied RNA arrays to identify apoptosis genes regulated by RA in breast cancer cells. The TNF and TNF receptor superfamily were upregulated and were involved in several programmed cell death signaling pathways [97]. 

RA promoted apoptosis in combination therapy. Aslıhan et al. showed that RA enhanced caspase-3 activity and synergized with siRNA to inhibit heat shock protein 27 (HSP27), which directly induced apoptosis in human glioma cells [111]. Mucin 1 (MUC1) attenuated mitochondrial apoptotic factors and conferred resistance to cytarabine, gemcitabine, and cisplatin [112]. RA combined with a MUC1 inhibitor enhanced the inhibition of protein glycosylation-related enzymes. Combination therapy induced apoptosis-related proteins, including p53, BAX, BCL-2 associated agonist of cell death (BAD), and caspases-3, -8, and -9 [113]. Docking studies showed that RA possesses a good binding affinity to the p53 protein [114].

### 5.5. Suppression of Multidrug Resistance (MDR) Proteins

The *ABCB1* gene and its functions encode MDR1/P-glycoprotein (P-gp) as an energy-dependent drug pump [115]. P-gp-mediated tumor resistance is combined with drugs, and the intracellular drugs are pumped extracellular through an ATP-dependent pathway, reducing intracellular drug concentration. RA has a remarkable sensitization to radiotherapy and chemotherapy [116] and has been observed as an inhibitory effect on P-gp in tumor therapy combined with DOX, DDP, and gemcitabine, leading to a sensitization effect on chemotherapy in gastric, breast, non-small-cell lung, and pancreatic cancers [11,117,118,119]. Studies indicated that MUC1 induces acquired chemoresistance by upregulating P-gp [120,121]. RA reduced MUC1 to sensitize chemotherapy in gastric cancer [113,122]; however, whether RA mediates P- gp through MUC1 requires further study.

### 5.6. Suppression of Glycolytic Pathway

Differentially expressed proteins after RA intervention in HCC were detected by proteomics analysis. Glycolysis and gluconeogenesis were significantly downregulated after RA intervention according to KEGG pathway enrichment. Inhibition of glycolysis reduced ATP production and inhibited the proliferation of HepG2 cells [123]. The Warburg effect and hypoxia-inducible factor 1 (HIF-1) strengthen energy metabolism, free radical accumulation, and chronic inflammation, promoting tumor angiogenesis and survival [124]. RA can mitigate IL-6/STAT3 and HIF-1α against the Warburg effect in gastric carcinoma and CRC [125,126].

### 5.7. EMT Inhibition

Invasive tumor cells exhibit characteristics associated with EMT, including mesenchymal cell morphology, loss of cell adhesion, upregulation of cell mobility, and expression of mesenchymal cell feature proteins [127]. RA regulated EMT-related proteins and inhibited tumor cell invasion [107]. RA promoted EMT through the upregulation of E-cadherin, inhibition of N-cadherin, and the concomitant inhibition of matrix metalloproteinases (MMPs), resulting in impaired invasive ability in osteosarcoma, pancreatic cancer, and CRC [108,128,129]. Studies suggested that RA suppressed the expression of Zinc finger E-box binding homeobox 1, snail1, and twist1, inhibited EMT, and increased chemosensitivity [117].

### 5.8. Anti-Angiogenesis and Metastasis

Both in vivo and in vitro studies have shown that RA has the ability to inhibit invasion and metastasis. First, RA can inhibit invasion ability through MMPs. The central role of MMPs in cancer metastasis is the degradation and remodeling of the extracellular matrix (ECM), which facilitates invasion and metastasis through peripheral cancer tissues. ECM-degrading proteolytic enzymes such as MMP-1, -2, -13, and -14 are involved [130]. Furthermore, the role of MMPs in promoting angiogenesis also promotes tumor progression and metastasis. MMP-9 regulates vascular endothelial growth factor (VEGF) translocation into cells to enable an angiogenic switch [131]. RA inhibited the expression of MMP-2 and MMP-9 and cell invasive ability in several tumor cell lines and attenuated lung metastasis of CRC in a mouse model [60,100,107]. The downregulation of MMP-2 and MMP-9 by RA as a Fyn inhibitor in treating HCC and glioma suppressed tumor invasion and migration [10,109]. Downregulation of AKT phosphorylation with repression by MMPs contributed to the suppression of tumor invasion ability [101,108]. Studies found that RA upregulated miRNAs; miR-506 and miR-1225-5p targeted the 3’ untranslated regions of MMPs to inhibit EMT and tumor metastasis [128,129].

Second, RA inhibits tumor metastasis through VEGF and IL-8 pathways. In vivo studies reported that RA inhibited lung metastasis and bone metastasis of breast cancer [60,132]. Downregulation of VEGF is also the most frequently reported target of RA treatment for metastasis [88,89,133]. Activation of EGFR and VEGF receptors promoted the expression of MMPs and VEGF through intracellular signaling cascades and inhibited the formation of metastatic lung nodule formation [60,132,134]. Huang et al. showed that ROS generation promoted VEGF expression and IL-8 release [134]. In addition, RA inhibited breast cancer metastasis by suppressing IL-8 through the NF-κB ligand/TNF receptor superfamily member 11a/osteoprotegerin pathway [132]. Nevertheless, the role of RA against metastasis requires further study. 

## 6. Prevention of RA in Tumorigenesis

**Figure 2:**
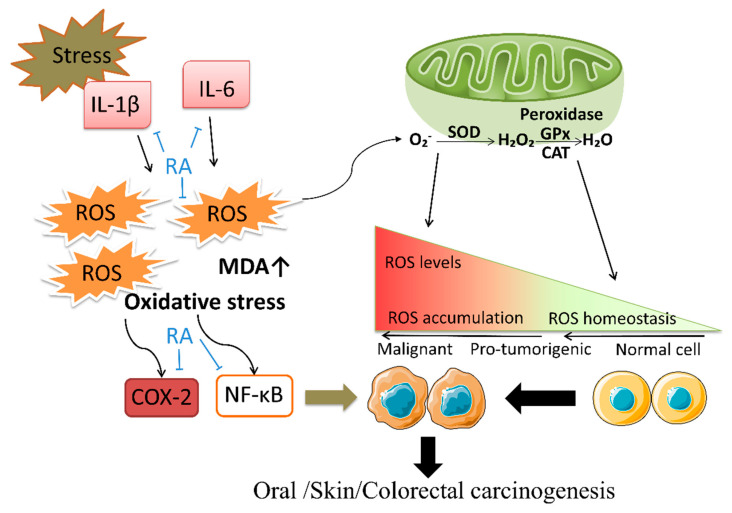
Mechanisms of tumorigenesis prevention effects of RA. When cells undergo oxidative stress or inflammatory factors (ILs), ROS and MDA are accumulated. The intracellular peroxidases SOD dismutate superoxide anion to H_2_O_2_, then GPx and CAT catalyze the decomposition of H_2_O_2_ into H_2_O to maintain cellular ROS homeostasis. When cells are continuously stimulated by ROS, accompanied by the activation of NF-κB and COX-2, the normal cells can abnormally proliferate, differentiate, and escape apoptosis, leading to tumorigenesis. RA inhibits the secretion of IL-1β and IL-6, the expression of NF-κB and COX-2, and downregulates the content of ROS and MDA.

Antioxidation and free radical scavenging are the crucial functions of RA to prevent tumorigenesis. RA upregulated activity of SOD, CAT, glutathione (GSH), and GPx and downregulated thiobarbituric acid reactive substances and malondialdehyde (MDA) [73,74]. Sufficient evidence on the prevention of CRC is related to the potent antioxidant effect of RA on tissues during intestinal epithelial absorption to prevent polyps and tumorigenesis. Common inducers in animal models of colorectal cancer are DMH, AOM and dextran sodium sulfate (DSS). AOM and DMH can become carcinogenic through DNA alkylation, promoting the mispairing of bases. DSS is a synthetic sulfated polysaccharide, and its use alone was shown to cause colonic inflammation in mice, while a combination of AOM and DSS stably induced inflammatory colorectal cancer [135]. In colorectal carcinogenesis rat or mouse models, RA reduced the formation of aberrant crypt foci (ACF) and eliminated the progression of colitis-associated colon cancer [9,74,76,92]. The potential prevention of CRC by RA was mainly attributed to three aspects. First, the excellent antioxidant effect of RA could enhance antioxidant enzyme activity, including SOD, CAT, GSH, and GPx. In addition, RA attenuated DMH-induced upregulation of cytochrome P450 (CYP450) [74,77]. Second, RA inhibited the release of TNF-α, IL-6, and COX-2 pro-inflammatory factors [76,79]. The anti-inflammatory effect of RA has been related to the inhibition of TLR4/NF-κB and STAT3 [9]. Third, RA can reduce DNA damage against ACF formation [92]. A model of spontaneous CRC, C57BL/6J-*Apc*^Min/+^ mouse, was shown to be related to familial adenomatous polyposis-derived tumorigenesis. RA decreased the numbers of large adenomas (>3 mm) in C57BL/6J-*Apc*^Min/+^ mouse [136]. Skin carcinogenesis is primarily caused by exposure to UV in sunlight, which induces oxidative stress, and the formation of photoproducts and lesions in DNA. Impaired DNA repair may lead to mutagenesis and carcinogenesis [137,138]. RA ameliorated ROS generation, MDA content, and DNA damage in DMBA/TPA-induced skin papilloma mouse model and UV-irradiated keratinocytes [20,24,70,139]. RA downregulated NLRP3 and IL-1β production via the Nrf2/HO-1 antioxidant system [78]. Overall, the occurrence of skin cancer is related to UV exposure and oxidative stress, and the above effects of RA can effectively resist skin carcinogenesis. RA prevents oral cancer due to its antioxidant effects [73,140], as illustrated in Figure 2 and Table 2.

## 7. The Therapeutic Effect of RA on Cancer

**Figure 3:**
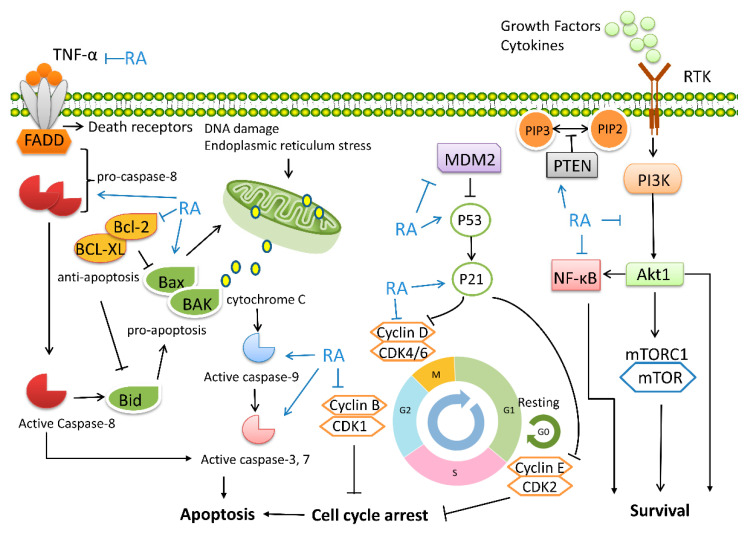
The key mechanisms of anti-tumor effects of RA. Extrinsic apoptosis: RA induces extrinsic apoptosis by upregulating TNF-α and caspase-8. Endogenous apoptosis: RA inhibits BCL-2 and promotes the expression of BAX, caspase-3, -7, and -9 to mediate endogenous apoptosis. P53 pathway and cell cycle: RA inhibits MDM2 expression and promotes p53, p21 expression mediates apoptosis. P21 inhibits Cyclin D and Cyclin E to induce cell cycle arrest, and RA inhibits Cyclin B, Cyclin D, and Cyclin E to mediate cell cycle arrest and promote apoptosis. PI3K/AKT and NF-κB pathway: RA promotes the expression of tumor suppressor gene PTEN, inhibits PI3K expression, AKT phosphorylation, NF-κB expression, and p65 phosphorylation, which induces apoptosis through the inhibition of cell survival-related pathways.

RA inhibited several solid and hematologic tumors by inducing cell cycle arrest and apoptosis, and inhibiting EMT and tumor metastasis. Studies on the anti-tumor effects of RA through in vitro and in vivo models are summarized in Figure 3 and Table 3. In the case of glioma, RA was reported to promote apoptosis-related protein and exerted cytotoxicity in several glioma cell lines with an IC_50_ value ranging between 200 and 400 µM for 48 h [10,111]. RA induced cell apoptosis and inhibited the migration of oral cancer cells in vitro [100]. RA regulated apoptosis-related genes and changed the methylation pattern via DNA methyltransferases 1 (DNMT1) for breast cancer chemoprevention [97,141]. In addition, RA inhibited breast-derived bone metastases by suppressing IL-8 [132]. RA suppressed the viability of two gastric cancer cell lines at a lower IC_50_ concentration of 240 µM; meanwhile, RA suppressed tumor growth in gastric tumor-bearing mice by inhibiting of the Warburg effect [122,125]. RA was shown to possess a wide range of applications in the treatment of HCC, including induction of apoptosis, and inhibition of tumor growth and metastasis. Meanwhile, RA demonstrated little effects on the proliferation and morphology of normal human astrocytes cells [109]. Treatment of RA mediated the upregulation of caspase-3, -8, and -9 and inhibited BCL-2 expression to induce apoptosis in different HCC cell lines [19,106]. The downregulation of PI3K/AKT and glycolytic pathway by RA inhibited the cell proliferation and tumor growth of HCC [109,110,123]. Furthermore, RA analogue-11 is a synthesized RA analogue, which promoted apoptosis via the EGFR/AKT/NF-κB pathway in gastric cancer cells [142]. RA also inhibited VEGF expression and EMT to attenuate tumor invasion of HCC in vitro, but more in-depth evidence in in vivo studies is required [88,106,110]. Pancreatic cancer is a highly lethal disease and the fourth leading cause of cancer-related deaths worldwide [143]. RA was shown to induce apoptosis and inhibit pancreatic cancer invasion and proliferation in vitro and suppressed tumor growth in vivo [98,129]. CRC is the third most common cause of cancer-related deaths worldwide, with tumor metastasis occurring in approximately 45% of patients [144]. In CRC, RA demonstrated the potential to withstand CRC metastasis. A reduction in lung metastasis was observed in mice model after RA treatment [107]. Meanwhile, RA can downregulate EMT and MMPs to inhibit the invasion and migration ability of several CRC cell lines [60,107,128]. Inflammation affects cytokine receptor-mediated signaling pathways that mediate CRC tumor progression, including the TNF, IL-1, IL-6, and NF-κB pathways. Moreover, therapy-induced death of CRC cells can induce the production of TNF, IL-17, and IL-6 to save the remaining cells [145]. Therefore, avoiding the inflammatory response could help treat CRC. In this regard, RA was shown to suppress CRC inflammation by impairing the IL-6/STAT3 and NF-κB pathways [60,126]. In solid tumors, RA has also shown anti-tumor effects on ovarian cancer, cervical cancer, prostate cancer, and osteosarcoma, and the specific mechanisms are shown in Table 3. Through transcriptome sequencing analysis, RA methyl ester accelerated apoptosis in DDP resistant ovarian cancer cell line through inhibitory of Forkhead box M1 (FOXM1) [146]. And RA methyl ester also enhanced DDP sensitivity against cervical cancer by inhibiting mTOR/ribosomal protein S6 kinase β-1 (S6K1) pathway [147]. For hematological tumors, RA reported to induce cytotoxicity against multiple myeloma (MM) by inhibiting mitochondrial activity [148]. RA promoted apoptosis in leukemia cells by inhibiting NF-κB and ROS production [87,90], and the IC_50_ values of RA-treated normal lymphocytes were 1.7- to 5-fold higher than that of ALL cells [90].

## 8. Chemosensitivity Effect of RA on Tumor Therapy

Tumor resistance to chemotherapy is a significant cause of treatment failure, and has led to research on chemotherapeutic drug sensitizers. DDP is a platinum coordination complex, which can inhibit the DNA replication of cancer cells and damage the cell membrane structure. It is cytotoxic and more sensitive to fast proliferation cells, such as cancer cells. Therefore, it is widely used in the anti-tumor treatment of solid tumors [149]. Platinum-based drugs are used in the first-line treatment of lung, liver, and ovarian cancer. Four studies showed that RA increased the sensitivity of malignant tumor cells to DDP. RA downregulated MDR1 to increase the sensitivity of DDP in treating lung cancer [11]. The combination of RA and DDP induced G2/M phase arrest and apoptosis in renal cancer cells [99]. RA inhibited melanin synthesis and increased DDP sensitivity by inhibiting the ADAM17/EGFR/AKT/glycogen synthase kinase-3β (GSK3β) axis in melanoma [101]. In addition, RA showed synergistic anti-proliferation effect with DDP on ovarian cancer cells [18].

In breast cancer treatment, DOX and paclitaxel are used as sequential chemotherapy regimen [150]. RA mediated the sensitivity of DOX and paclitaxel by regulating p53 pathway and inducting apoptosis [89,118]. The first-line chemotherapy drug for gastric cancer treatment is 5-fluorouracil (5-FU). RA was shown to enhance chemosensitivity to 5-FU by increasing Forkhead box O4 (FOXO4) [151]. DOX is a chemotherapeutic drug used for gastric cancer treatment and RA was shown to reverse the resistance of SGC7901/Adr cells to DOX by inhibiting MDR1 [119]. In addition, RA cooperated with the anti-MUC1 antibody to promote apoptosis in human gastric carcinoma cells [113]. Although the recommended treatment of HCC is surgery, radiotherapy, and interventional therapy, chemotherapy and molecular-targeted therapy are still the main treatment options for advanced HCC. Combination therapy using RA and DOX can enhance DNA damage and BAX/BCL-2 ratio in HCC [93]. RA synergistically increased cytotoxicity and proteasome inhibition induced by MG132 in HCC [152]. RA also enhanced the efficacy of gemcitabine through the downregulation of multidrug resistance-associated protein 4 (MRP-4) and MRP-5 in Panc-1 pancreatic cancer cells [117]. Treatment with all-trans retinoic acid (ATRA) induced the differentiation of leukemia cells and increased the complete response rate of acute promyelocytic leukemia (APL) [153]. More than 80–90% of APL are expected to be cured with a therapeutic regimen based on ATRA and arsenic trioxide [154]. RA potentiated ATRA-induced macrophage differentiation in APL cells [155]. Then, RA synergistically inhibited DNA synthesis to potentiated the anti-proliferative effect of Ara-C [81]. In addition, RA can increase the sensitivity of physical therapy. Combining blue light and RA was shown to effectively decrease the cell proliferation of HNSCC [80]. RA specifically sensitized radiation to induce apoptosis in metastatic melanoma [156]. Using RA for synthesizing AuNPs plays an active role on the treatment of breast cancer [157].

Some RA-rich herbs have been used in adjuvant chemotherapy in ethnomedicine to sensitize cancer cells to conventional drugs and enhance their effects at minimal doses. Subsequent studies confirmed that RA indeed increased the sensitivity of commonly used chemotherapeutic drugs, including DDP, paclitaxel, 5-Fu, DOX, and Ara-C. The molecular targets involved in chemosensitization are displayed in Table 4.

## 9. Conclusions

Substantial evidence has been proven the potential benefits of RA and RA-enriched plants as drug candidates for the prevention and treatment of cancer. Among RA-rich plants, rosemary, basil, and *Perilla frutescens* (L.) Britt are potential anti-tumor plants as dietary supplements. In this review, antioxidative and anti-inflammatory effects of RA prevent tumorigenesis, and oral RA is a potential application to prevent CRC. RA exerts anti-tumor effects by inhibiting tumor cell proliferation and EMT, inducing cell cycle arrest and apoptosis, in which PI3K/AKT, NF-κB, IL-6/STAT3, p53, VEGF, and glycolysis pathways are involved. Inhibition of MDR protein by RA increases chemosensitivity in tumor therapy. In tumor therapy, RA is widely used in the treatment of digestive system tumors, including HCC and CRC. In addition, RA can increase the sensitivity of DDP and DOX drugs in the treatment of solid tumors. To improve the oral bioavailability of RA, modification of excipients, encapsulation using cyclodextrins, drug delivery systems, and derivatives of RA are promising candidates. This review provides a theoretical basis for the use of RA in the prevention and treatment of cancer. However, RA is worthy of further investigation based on high-throughput methods and clinical studies. It is expected to become one of the promising methods for preventing and treating cancer in the future.

## Figures and Tables

**Table 2:** Summary of tumorigenesis prevention effects of RA.

Disease	Model	Treatment	Outcome	Ref
Colorectal carcinogenesis	Wistar male rats given DMH orally 20 mg/kg, once a day	RA 10mg/kg, once a day	Inhibited the carcinogenic effect through circulatory antioxidant enzymes (SOD↑, CAT↑, GSH↑, and GPx↑)	[75]
Colorectal carcinogenesis	Male albino wistar rat given DMH 20 mg/kg subcutaneously for 4 weeks	RA 2.5, 5, and 10 mg/kg	Reduced the polyp incidence through CYP450↓, lipid peroxidation↓, SOD↑, CAT↑, GPx↑, and GSH↑.	[74]
Colorectal carcinogenesis	Wistar rats with subcutaneous injection of 40 mg/kg DMH for 2 weeks	RA 4, 8 and 16 mg/kg body weight	Reduced DNA damage and frequency of the formation of ACF	[92]
Colorectal carcinogenesis	Male Wistar rats with subcutaneous injection of DMH 20 mg/kg.	Oral RA 5mg/kg body weight 30 weeks in total	Inhibited the tumor formation and reduced expressions of TNF-α, IL-6, and COX-2, and increased SOD, CAT, GPx, and TBARS	[76]
Colorectal carcinogenesis	Male Wistar rats with subcutaneous injection of DMH 20 mg/kg for 15 weeks	Daily RA 5mg/kg orally	Protected the activity of antioxidant enzymes (CYP450↓ and CYP4502E1↓) and reduced the formation of ACF	[77]
Colorectal carcinogenesis	Male Sprague-Dawley rats intraperitoneally injected with 15 mg/kg AOM once a week for 4 weeks	RA 5 mg/kg orally per day	Increased the total antioxidant status, and decreased the expression of IL-6 and total oxidant status	[79]
Colorectal carcinogenesis	Male BALB/c mice with oral administration of AOM5-ASA 75 mg/kg/day intraperitoneally for 7 days, then supplied drinking water containing 1–2% DSS for 49 days	RA 30 mg/kg/day orally	Inhibited TLR4 mediated the activation of NF-κB and STAT3 and eliminated the progression of colitis-associated colon cancer	[9]
Colorectal carcinogenesis	APC10.1 cells; C57BL/6J-*Apc*^Min/+^ mouse model	RA 100 µM; 0.3% RA in the diet, 360 mg/kg per day	Decreased numbers of large adenomas (>3 mm)	[136]
Skin carcinogenesis	DMBA/TPA induced skin papilloma mouse model	Topical application RA 1.35 mg/mouse	Inhibited MDA, chemokines and arachidonic acid and prevented DNA from oxidative damage	[20]
Skin carcinogenesis	HaCaT cells exposed to UVA	RA 2.7–18 mg/mL	Attenuated ROS generation and DNA damage in UVB-irradiated keratinocytes by LBE	[24]
Skin carcinogenesis	HaCaT cells exposed to UVB	RA 2.5 or 5 µM	Downregulated the inflammasome components (NLRP3 and IL-1β production) via Nrf2/HO-1 antioxidant system and prevented skin changes caused by UVB	[78]
Skin carcinogenesis	B16 melanoma cells; Female albino Swiss mouses exposed to UVA light 3 times a week, total 100 times	2% RA in the diet to rats; Cell administration RA at 1 mg/mL	RA increased the Tyr activity in vitro. Oral RA inhibited skin changes caused by UVA exposure (skin photocarcinogenesis)	[70]
Oral carcinogenesis	0.5% DMBA liquid paraffin treated on left buccal pouches of golden Syrian hamster model for 14 weeks	RA orally 100 mg/kg	Suppressed oral carcinogenesis through upregulation of SOD, CAT, GSH, GPx and downregulation of TBARS and BCL-2	[73]
Oral carcinogenesis	Male Syrian hamster intravenous injection of 0.5% DMBA	RA 1.3 mg/15mL	Reduced the intensity and invasiveness of the tumor	[140]
Tumor angiogenesis	Human umbilical vein endothelial cells (HUVECs)	RA 50, 100 and 200 mM	Suppression of ROS generation and downregulation the release of VEGF and IL-8	[134]

**Table 3:** Summary of anti-tumor effects of RA.

Disease	Model (IC_50_)	Treatment	Outcome	Ref
Glioma	U251 and U343 glioma cells	RA 100, 200, and 400 µM	Inhibited BCL-2 and promoted the expression of BAX and cleaved caspase-3 protein, and downregulated PI3K/AKT/NF-κB signaling pathway through targeting Fyn.	[10]
Glioma	U-87 MG cells (IC_50_ for 48 h:373.48 μM)	RA 80 and 215 µM	Inhibited the expression of HSP27 and enhanced the activity of caspase-3	[111]
Oral cancer	SCC-15 cells	RA 10, 20, and 40 µM	Increased the expression of cleaved caspase-3 and BAX/BCL-2 ratio, induced G2/M cell cycle arrest, and inhibited migration through downregulation of MMP-2 and MMP-9	[100]
Breast cancer	MDA-MB-231 (IC_50_ for 48 h: 321.75 ± 9.75 uM) and MDA-MB-468 cells (IC_50_ for 48 h: 340.45 ± 7.57 uM)	RA 125 and 250 µM	Induced G0/G1 cell cycle arrest and apoptosis through regulation of apoptosis-related genes (*HRK*↑, *TNFRSF25*↑, *BNIP3*↑, *TNF*↑, *GADD45A*↑, *BNIP3*↑, *TNFSF10*↓, *BIRC5*↓ and *TNFRSF11B*↓)	[97]
Breast cancer	MCF7 cell line	RA 20 and 40 µM	Regulated the methylation pattern via DNMT1 for chemoprevention of cancer	[141]
Breast-derived bone metastases	MDA-MB-231BO human bone-homing breast cancer cells (IC_50_: 118.04 µg/mL)	RA 7.5, 15, 30, and 60 µg/mL	Inhibited the metastasis of breast cancer by suppression of IL-8 through NF-κB ligand/ TNF receptor superfamily member 11a /osteoprotegerin pathway	[132]
Gastric cancer	MKN45 cells (IC_50_ for 24 h: 240.2 μM); MKN45 cells injected into BALB/c-nude mice	RA 60, 120.1, and 240.2 µM; RA 2 mg/kg injected intraperitoneally for 14 days	Inhibited Warburg effect (glucose consumption, lactate generation, and HIF-1α) through downregulation of IL-6/STAT3 pathway	[125]
Gastric cancer	CRL-1739 cells (IC_50_ for 24 h: 240 μM)	RA 100 and 200 μM	Inhibited the expression of MMP-9, TIMP-1, MUC1, Tn antigens and T antigens, increased the expression of collagen I	[122]
Gastric cancer	GES-1 (IC_50_ for 24 h: 289.425 ± 0.854 μmol/L) and SGC-7901 cells (IC_50_ for 24 h: 73.299 ± 2.011 μmol/L)	RA analogue-11 10, 20, and 40μmol/L	Promoted apoptosis via the EGFR/AKT/NF-κB pathway in gastric cancer cells.	[142]
HCC	HepG2 cells	RA 5 and 10 µg/mL	Induced apoptosis through increasing the mRNA levels of Jun, Jun-B, Fos-B, BAX and caspase-8, and decreased BCL-2 mRNA expression	[19]
HCC	H22 tumor-bearing mice	Intraperitoneal injection of RA 75, 150, and 300 mg/kg	Inhibited inflammatory cytokines (IL-1β, IL-6, TNF-α, TGF-β), angiogenic factors (VEGF) and phosphorylation of p65. The tumor inhibition rates in different concentrations of RA (39.03%, 42.98%, and 48.24%)	[88]
HCC	HepG2 cells (IC_50_ for 48 h: 33 ± 0.74 μg/mL)	RA 6.25, 12.5, 25, 50, and 100 µg/mL	Inhibited the expression of GLUT-1 and HK-2 to suppress the glycolytic pathway.	[123]
HCC	HepG2 cells	RA 7, 14, and 28 µM	Induced apoptosis (caspase-3↑, caspase-9↑ and BAX/BCL-2 ratio↑), inhibited migration, and invasion	[106]
HCC	HepG2 cells	RA 100, 200, and 400 µM	Reduced the expression of MMP-2, MMP-9, and BCL-2, promoted the expression of BAX and Caspase-3, and downregulated PI3K/AKT/NF-κB signaling pathway through targeting Fyn.	[109]
HCC	SMMC 7721 cells; Tumor bearing model of nude mice	RA 20, 50, and 100 µmol/L; RA 5, 10, and 20 mg/kg for 5 days	Downregulated PI3K/AKT/mTOR signaling pathway to induce apoptosis, inhibited EMT in vitro and tumor growth in vivo	[110]
Pancreatic cancer	PANC-1, PATU-8988, MIA PaCa-2 and BxPC-3 cells; Tumor bearing model of nude mice (MIA PaCa-2 cells)	RA 100, 200, 300, 400, and 500 μM; Orally 50 mg/kg RA 50 mg/kg orally for 30 days	Enhanced proteasome-mediated degradation of Gli1 and inhibited the expression of downstream VEGF, Cyclin D1 and snail1. Induced apoptosis and inhibited invasion and proliferation in vitro; Suppressed tumor growth in vivo	[98]
Pancreatic cancer	Panc-1 (IC_50_ for 24 h: 104.2 ± 4.5 μM) and SW1990 cells (IC_50_ for 24 h: 118.9 ± 6.7 μM); Nude mice injected subcutaneously into Panc-1 cells	RA 100 µM; 10 and 50 mg/kg orally for 30 days	Inhibited mRNA expression of MMP2 and MMP16 via miR-506; Inhibited tumor growth in the xenograft mice model.	[129]
CRC	HCT15 and CO115 cells	RA 10, 50, and 100 µM	Inhibited cell proliferation through inhibitory of phospho-ERK in HCT15	[35]
CRC	HCT8 (IC_50_: 298.1 μM), HCT116 (IC_50_: 319.8 μM), Ls174-T (IC_50_: 539.4 μM), and Lovo (IC_50_: 576.3 μM) cells	RA 0, 75, and 150 µM	Inhibited IL-1β, TNFα, IL-6, and STAT3 against Warburg effect	[126]
CRC	CT26 and HCT116; BALB/c mice inoculated with CT26 via the lateral tail vein	RA 50, 100, and 200 µM; oral injection of RA (100 mg/kg/day) for 14 days	Induced G0/G1 cell cycle arrest and apoptosis (caspases↑, Bcl-XL↓, and BCL-2↓), inhibited EMT and invasion via AMPK phosphorylation; Reduced lung metastasis of CRC cells	[107]
Colon carcinoma Lung cancer	Ls174-T human colon carcinoma cells. Lewis lung carcinoma (LLC) cells injected into C57BL/6 mice	RA 37.5, 75, 150, and 300 µg/mL in vitro; RA 1, 2, and 4 mg/kg intraperitoneal injection for 20 days	Inhibited the activities of EGFR and VEGFR, and then suppressed the nuclear translocation of NF-κB and activity of p-AKT and p-ERK resulting in downregulation of the mRNA and protein expression of MMP-2, MMP-9, and VEGF in vitro. Inhibited the formation of metastasis nodules.	[60]
CRC	HT-29 cells	RA 50, 100, and 200 µM	Inhibited EMT (E-cadherin**↑**, N-cadherin**↓**, MMP-1, -3, and -9**↓**) via the p38/AP-1 signaling	[128]
Ovarian cancer	OVCAR-3 cells	RA 10, 40, and 160 µM	Regulated the expression of lncRNA MALAT-1, inhibited cell migration and induced apoptosis.	[94]
**Ovarian cancer**	SKOV-3, TOV-21G and DDP resistant daughter line TOV/CisR	RA methyl ester 40 µM; DDP 5µM; combination therapy	Accelerated apoptosis in DDP resistant ovarian cancer cell line through inhibitory of FOXM1	[146]
**Cervical cancer**	HeLa and SiHa cells	RA methyl ester 80 µM; DDP 5µM; combination therapy	Exerted apoptosis effects against cervical cancer by inhibiting mTOR/S6K1 pathway	[147]
Prostate cancer	PC-3, DU145 cells	RA 200 µM	Induced G0/G1 cell cycle arrest (Cyclin D1↓, Cyclin E↓, CyclinB1↓ and p21↑) and apoptosis, enhanced transcription of p53 by inhibition of HDAC2.	[96]
Osteosarcoma	U2OS (IC_50_ for 48h: 28 ± 1.14 μg/mL) and MG63 (IC_50_ for 48h: 25 ± 1.37 μg/mL) osteosarcoma cells.	RA 12.5, 25, and 50 µg/mL	Induced apoptosis (caspase-3, -8, and -9↑ and BAX/BCL-2 ratio↑), inhibited EMT and invasion (MMP-2↓, MMP-9↓) through DJ-1 mediated upregulation of PTEN and downregulation of PI3K/AKT	[108]
MM	ARH-77 cells	RA 50, 100, and 200 µM	Exerted cytotoxic effects and decreased the mitochondrial activity	[148]
Leukemia	U937 cells using TNF- α 10 ng/mL induced oxidative stress	RA 60 µM	Reduced NF- κB and ROS production, promoted apoptosis	[87]
Acute lymphoblastic leukemia	CCRF-CEM (IC_50_ for 48h: 14.6 ± 1.58 μM) and CEM/ADR5000 (IC_50_ for 48h: 44.5 ± 5.3 μM) cells	RA 15, 30, and 60 µM	Targeted IKK-β to inhibit NF-κB signaling pathway, caused disruption of MMP and cell adhesion and promoted caspase-independent cell death	[90]

**Table 4:** Summary of RA as chemosensitizers in tumor therapy.

Disease	Model	Treatment	Outcome	Ref
Lung cancer	A549 and A549/DDP (DDP resistance) cells	RA 10, 15, 20, and 40 µg/mL; DDP 1 µg/mL; combination therapy	Inhibited proliferation and invasion, and enhanced chemosensitivity to DDP based on downregulation of MDR1 mRNA expression	[11]
Renal cancer	786-O cells	RA 25, 50, and 100 µM; DDP 5µM; combination therapy	Induced G2/M phase arrest and apoptosis in renal cancer cells.	[99]
Ovarian cancer	A2780 and DDP resistant daughter line A2780CP70	RA 2.5, 5, and 10 g/mL	Showed synergistic anti-proliferation effect with DDP on A2780 cells	[18]
Melanoma	A375 cells	RA 50, 100, and 200 µg/mL; DDP 8 µM; combination therapy	Inhibited cell proliferation, invasion, and melanin synthesis, and increased apoptosis and DDP sensitivity via inhibitory of ADAM17/EGFR/AKT/GSK3β axis	[101]
Breast cancer	Female Swiss albino mice with intradermal injection of 0.1 mL Ehrlich ascites carcinoma	Oral RA 50 mg/kg; Paclitaxel 10 mg/kg/three times weekly intraperitoneally; combination therapy	Exerted chemo-preventive in combination with paclitaxel, suppressed NF-κB, TNF-α, and VEGF, increased in apoptotic markers p53, caspase-3, and BAX/BCL-2 ratio	[89]
Breast cancer	MCF-7 cells	RA 1.5, 15, or 50 µM; DOX 0.2 µM; combination therapy	Decreased the MDM2 gene expression and potentiated the effect of DOX	[118]
Gastric cancer	AGS cells	RA 100 and 200 µM; Anti-MUC1 antibody 5 µg/mL combination therapy	Inhibited the expression of MUC1, BCL-2, Tn antigens and T antigens, increased the expression of caspase-9, BAX, and BAD	[113]
Gastric cancer	SGC7901/Adr cells (DOX resistance)	RA 2.4 and 12 µM	Reversed the MDR of SGC7901/Adr cells, increased sensitivity to DOX and Rh123 through downregulating the expression of MDR1 transcript levels	[119]
Gastric cancer	SGC7901 and SGC7901/5-Fu (5-Fu resistance) cells	RA 15 µg/mL; 5-Fu 50 µg/mL; combination therapy	Enhanced chemosensitivity to 5-Fu, increased FOXO4 by downregulating miR-6785-5p and miR-642a-3p	[151]
HCC	HepG2 and Bel-7402 Cells	RA 25, 50, and 100 µg/mL; DOX 0.4 µg/mL; combination therapy	Enhanced DNA damage and apoptosis (BAX/BCL-2 ratio↑)	[93]
HCC	HepG2 cells	RA 10, 100, and 1000 mM; MG132 1 µM; combination therapy	Synergistically increased cytotoxicity, proteasome inhibition, autophagy, and apoptosis	[152]
Pancreatic cancer	Panc-1 cells	RA 10 and 20 µM; Gemcitabine 12.5 nM; combination therapy	Exerted anti-migration, pro-apoptosis effects and enhanced the efficacy of gemcitabine through downregulation of MRP-4, MRP-5, and Notch1 intracellular domain	[117]
APL	NB4 cells	RA 40 mM; ATRA 10 nM; combination therapy	RA potentiated ATRA-induced macrophage differentiation in APL cells and increased CCR-1, CCR-2, and ICAM-1 expression through activation of ERK and NF-κB	[155]
APL	HL-60 cells	RA 100, 125, and 150 µM; Ara-C 5, 10, and 20 nM; combination therapy	Synergistically inhibited DNA synthesis to potentiated the anti-proliferative effect of Ara-C	[81]
HNSCC	UM-SCC-1, UM-SCC-6, and OSC-2 cells	RA 80 µg/mL; Blue light 400–500 nm; 60 J/cm^2^, 2 min; combination therapy	Reduced EGFR activation and H_2_O_2_ production.	[80]
Metastatic melanoma	B16F10 cells	RA 20 and 40μM; RA combination with X-rays	Specifically sensitized radiation induces apoptosis of tumor cells	[156]

## Data Availability

Not applicable.

## Abbreviations

RA, Rosmarinic acid; CRC, colorectal cancer; DDP, cisplatin; HCC, hepatocellular carcinoma; DMBA, 7,12-dimethylbenz[a]anthracene; TPA, 12-tetradecanoylphorbol 13-acetate; ROS, reactive oxygen species; HPPR, Hydroxyphenylpyruvate reductase; HNSCC, head and neck squamous cell carcinoma; BAP, 6-benzylaminopurine; NAA, naphthalene acetic acid; MAPK, the mitogen-activated protein kinase; ERK1/2, extracellular signal-regulated kinases 1 and 2; PI3K, the phosphatidylinositide-3-kinase; MeJA, Methyl jasmonate; TAT, tyrosine aminotransferase; ALL, acute lymphoblastic leukemia; TiO_2_ NPs, titanium dioxide nanoparticles; IC_50_, half-maximal inhibitory concentration; RAS, rosmarinic acid synthase; SLN, solid lipid nanoparticles; DOX, doxorubicin; SOD, superoxide dismutase; CAT, catalase; DMH, 1,2-dimethylhydrazine; AOM, azoxymethane; GPx, glutathione peroxidase; EGFR, epithelial growth factor receptor; Ara-C, cytarabine; NLRP3, NOD-like receptor family pyrin domain containing 3; IL-1β, interleukin-1β; IL-6, interleukin-6; TLR4, Toll-like receptor 4; STAT3, signal transducer and activator of transcription 3; COX-2, cyclooxygenase-2; IKK-β, I-kappaB kinase-β; TNF-α, tumor necrosis factor-α; TGF-β, transforming growth factor-β; BAX, BCL-2 associated X; BCL-2, B cell lymphoma-2; PARP, poly (ADP-ribose) polymerase; HDAC2, histone deacetylases 2; Gli1, glioma-associated oncogene homolog 1; SYK, spleen tyrosine kinase; MARK4, microtubule affinity regulating kinase 4; MCM7, mini-chromosome maintenance complex component 7; BAX, BCL-2 associated X; mTOR, the mechanistic target of rapamycin; EMT, epithelial-mesenchymal transition; HSP27, heat shock protein 27; MUC1, Mucin 1; BAD, BCL-2 associated agonist of cell death; MDR, multidrug resistance; P-gp, P-glycoprotein; HIF-1, hypoxia-inducible factor 1; MMPs, matrix metalloproteinases; ECM, extracellular matrix; VEGF, vascular endothelial growth factor; GSH, glutathione; MDA, malondialdehyde; DSS, dextran sodium sulfate; ACF, aberrant crypt foci; CYP450, cytochrome P450; DNMT1, DNA methyltransferases 1; FOXM1, Forkhead box M1; S6K1, ribosomal protein S6 kinase β-1; MM, multiple myeloma; 5-FU, 5-fluorouracil; FOXO4, Forkhead box O4; MRP-4, multidrug resistance-associated protein 4; ATRA, all-trans retinoic acid.
